# The role of social attraction and social avoidance in shaping modular networks

**DOI:** 10.1098/rsos.231619

**Published:** 2024-02-28

**Authors:** Valéria Romano, Ivan Puga-Gonzalez, Andrew J. J. MacIntosh, Cédric Sueur

**Affiliations:** ^1^ IMBE, Aix Marseille Univ., Avignon Univ., CNRS, IRD, Marseille, France; ^2^ Center for Modelling Social Systems (CMSS) at NORCE, Kristiansand, Norway; ^3^ Wildlife Research Center, Kyoto University, Inuyama Campus, Inuyama, Japan; ^4^ Université de Strasbourg, CNRS, IPHC UMR 7178, 67000 Strasbourg, France; ^5^ Institut Universitaire de France, Paris, France

**Keywords:** group-living, social trade-off, interaction costs and benefits, behavioural variation, complex system, agent-based model

## Abstract

How interactions between individuals contribute to the emergence of complex societies is a major question in behavioural ecology. Nonetheless, little remains known about the type of immediate social structure (i.e. social network) that emerges from relationships that maximize beneficial interactions (e.g. social attraction towards informed individuals) and minimize costly relationships (e.g. social avoidance of infected group mates). We developed an agent-based model where individuals vary in the degree to which individuals signal benefits versus costs to others and, on this basis, choose with whom to interact depending on simple rules of social attraction (e.g. access to the highest benefits) and social avoidance (e.g. avoiding the highest costs). Our main findings demonstrate that the accumulation of individual decisions to avoid interactions with highly costly individuals, but that are to some extent homogeneously beneficial, leads to more modular networks. On the contrary, individuals favouring interactions with highly beneficial individuals, but that are to some extent homogeneously costly, lead to less modular networks. Interestingly, statistical models also indicate that when individuals have multiple potentially beneficial partners to interact with, and no interaction cost exists, this also leads to more modular networks. Yet, the degree of modularity is contingent upon the variability in benefit levels held by individuals. We discuss the emergence of modularity in the systems and their consequences for understanding social trade-offs.

## 1. Introduction

Understanding the link between individual behaviour and the organization and functioning of a group or population has long been central to behavioural and evolutionary biology [1]. Animals living in groups often interact non-randomly with individuals, leading to a large range of social structural patterns (i.e. who interacts with whom and how frequently). Social attraction and social avoidance, i.e. the aggregation or repulsion of individuals, are widespread behavioural strategies that individuals use to balance the costs and benefits of group-living. While some socio-ecological pressures like predator defence and access to reliable information lead to social attraction, others like resource competition and risk of pathogen transmission lead to social avoidance. But, how individuals manage the fitness trade-offs between attraction and avoidance has received little direct attention in the biological literature [2–4]. Recent studies have investigated this issue in the context of pathogen and information transmission [5–7], indicating that contagions may affect the evolution of social interactions [8]. This has been shown to be dependent on the type of contagion observed [9], social learning [10] and pathogen virulence [6]. Therefore, in nature, myriad factors influence how individuals manage opposing forces in social interaction.

Among these factors, studies highlight that individual decisions about with whom to interact appear sensitive to individual differences in their status [11–14]. When an individual shows signals of being a potentially beneficial or costly interaction partner, it not only affects its own dyadic interactions but also influences the structure of the overall group network [15,16]. For example, individuals who acquire environmental information quickly and use it frequently in front of others, thereby providing interaction benefits, become more central in their affiliative networks [17,18]. On the other hand, individuals showing clear signals of sickness, thereby demonstrating interaction costs, are frequently avoided [19,20]. This can significantly decrease the degree of connectedness between individuals in a group [21]. The growing body of evidence demonstrating plasticity in social network structure raises some key questions [2,22,23], such as to what extent these two mechanisms, social attraction and social avoidance, interact to influence the emergent properties of social networks under varying conditions.

To assess such behavioural flexibility, network metrics are useful measures that provide refined estimations of social interaction patterns. These so-called *network properties* can be classified at the individual level, for example, reflecting an individual's relative centrality in a network, or at the global level, such as when estimating a network's density or modularity. Among these global network properties, one has received considerable attention in the literature: *modularity*. Modularity is the extent to which a network is divided into differentiable subgroups [24], and is speculated to reduce the costs of connections between nodes (e.g. in neural networks [25]). In animal societies, it has been suggested that increased network modularity, which is typically associated with larger groups of vertebrates and invertebrates, might decrease costly social connections, such as those involving pathogen transmission [26,27]. Although the optimal subgrouping level that ‘breaks’ transmission chains is currently under discussion [28], a theoretical study working on about 2800 simulated networks and 41 real primate networks suggests that *network efficiency*—a measure of the ease with which an entity, such as a piece of information or an agent of infectious disease, can spread throughout a network [29]—peaks with intermediate levels of modularity [30].

**Figure 1.**
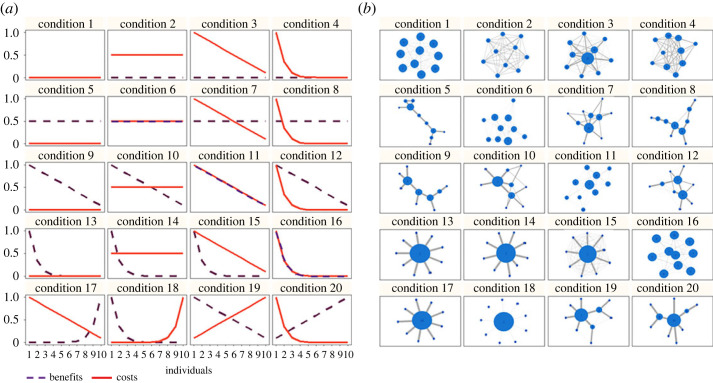
Schematic of the theoretical conditions under study (*a*) and the emergent social networks after 2000 interactions per individual (*b*). Networks are examples of model outputs with a group size = 10. In (*a*), the distribution of values assigned to benefits (purple dashed lines) and costs (red continuous lines) vary from 0 to 1 (*y*-axis) across individuals (*x*-axis: individuals' ID number, *N* = 10). The functions determining the values of benefits/costs were either linear (Y = 0; Y = 0.5; Y = (1/N) × ID; Y = 1 − (1/N) × ID − 1), where ID is the ID of the individual) or power-law distributed (*γ* = 10), and calculated using the unique ID number of individuals ranging from 1 to *N*. In (*b*), nodes (circles) represent individuals in the model (for *N* = 10 here) with their sizes directly related to the individual degree centrality coefficient (the higher the centrality, the larger is the size of the node). Network edges are undirected and weighted, such that pairs with higher association indices have thicker edges. Networks were built using the ‘igraph’ package in R [31].

In the present study, we investigated whether simple rules of social attraction and social avoidance—here modelled as individuals aiming to maximize social interactions with individuals holding benefits while minimizing costly interactions—leads to immediate topological consequences, specifically leading to a modular structure in social networks. Though it seems obvious that the presence of beneficial social interactions should lead to social aggregation, and the presence of costly social interactions to disaggregation, the extent to which these opposing forces concurrently influence the formation of social relationships has yet to be investigated. To achieve this goal, we developed an agent-based model in which individuals choose with whom to interact among a set of individuals that display honest signals about their status, beneficial and/or costly, to varying degrees. In this model, individuals are programmed to seek benefits (social attraction) while avoiding costs (social avoidance). Individuals are assigned values of *My-benefits* (hereafter termed ‘*benefits’* as used in our models) and *My-costs* (hereafter termed ‘*costs*’), which are varied systematically across conditions (conditions 1–20, figure 1*a*). We do not define a specific type of benefit or cost here, as our goal is to represent the myriad possibilities they could reflect. These theoretical systems represent fixed or slow-changing traits in a population, where costs and benefits essentially reflect the net effects (net-positive or net-negative) of interacting with a given social partner.

Our model was designed to reflect vertebrates that live in stable and cohesive groups, where fission–fusion mechanisms are absent or rare. We chose group sizes of 10, 30 and 70 because most of the populations that comprise cohesive groups with differentiated and individualized relationships that persist through time are roughly in this size range [32,33]. Therefore, we consider neither simple aggregations nor highly organized social insect groups. We purposely omitted social factors such as dominance rank [34] and environmental factors such as resource availability [35], which might also drive individual social preferences, so that we could create a parsimonious system that simply reflects the interactive influence of social attraction and social avoidance on network structure. Values of benefits and costs were also fixed during each simulation for parsimony, reflecting our goal of investigating the immediate topological consequences of individual decisions about with whom to interact. However, these values varied according to the tested conditions (figure 1*a*) because we aimed to observe the network properties that emerged. In addition to modularity, we chose to investigate network density (i.e. how well connected the whole network is [36]) and centralization (i.e. whether one or a few individuals monopolize(s) the interactions in the network [36]), as each provide a complementary view on network structure [37]. Biologically, and in the context of this manuscript, these measures reflect the preference of social interactions towards a few or several individuals in the group.

To make specific inferences and predictions, we first examined the conditions in which only costs (conditions 3 and 4) or only benefits (conditions 9 and 13) exist among individuals in the system. In conditions 3 and 4, we predicted the emergence of centralized, non-modular and high-density networks because individuals would prefer interacting with those showing low cost values. In conditions 9 and 13, we predicted the emergence of centralized, non-modular and low-density networks because individuals would prefer interacting with those having high benefit values. For conditions in which cost values (condition 2) or benefit values (condition 5) were evenly distributed across individuals, we predicted random networks as interactions might occur simply by chance, given our model rules.

With the above in mind, and related to the main aim of this study, we then investigated the emergent networks under conditions in which beneficial and costly individuals existed in the system simultaneously (conditions 6–8, 10–12, 14–20; figure 1*a*). In these conditions, we predicted the emergence of centralized, modular and low-density networks because individuals should develop social preferences for the most beneficial individuals while avoiding those that are most costly.

## 2. Material and methods

### 2.1. The optimal relationships model

This section describes the model (Process overview and scheduling) and the testing conditions (Testing conditions). A detailed description of the model according to the overview, design concepts and details (ODD) protocol [38], and the source codes of the model are given in the electronic supplementary material. The model was written in Netlogo v. 6.0 [39].

#### 2.1.1. Process overview and scheduling

The purpose of the model is to identify the type of immediate social structure (i.e. social network) that emerges from relationships that maximize beneficial interactions (e.g. social attraction towards informed individuals) and minimize costly interactions (e.g. social avoidance of infected individuals). Individuals are endowed with values of benefits and costs, which remain stable throughout the simulation and are assigned a number between 0 and 1 (where zero means 0% of benefits (or costs)) and one means 100% of benefits (or costs)). This is an honest-signalling system, meaning that individuals show external signals of the benefits and costs they uphold. The higher the value, the higher the benefit and/or cost.

At initialization, individuals have the same probability of interaction with every other individual, i.e. individuals are equally likely to select any given group member as an interaction partner (see §2.1.1.1, equation (2.1)). As they interact and perceive their associated benefits and costs, their probability of interaction changes. This subsequent probability of interaction is given by the weight of the relationship between the active and target individual: the stronger the weight, the higher the probability of interacting with that individual. Thereafter, individuals will increase or decrease the weight of their relationships over time according to whether they perceive costs and/or benefits from their partner (see §2.1.1.2). The amount of increase or decrease in the weight of the relationship is controlled by the parameters social-increase and social-decrease (table 2). After each social interaction, the weights of relationships, and thus the future probability of interaction, are updated (see §2.1.1.2). Note that the weights between individual *i* and all its group members sum to 1.

At each time step, all individuals are activated and forced to interact with one individual at a time; hence, the model represents stable group-living animals (no aggregates or fission–fusion societies), where individuals remain together most of time and thus socially interact. Different individuals may choose to interact with the same target individual, but each individual only chooses one partner with whom to interact at a time.

**Figure 2.**
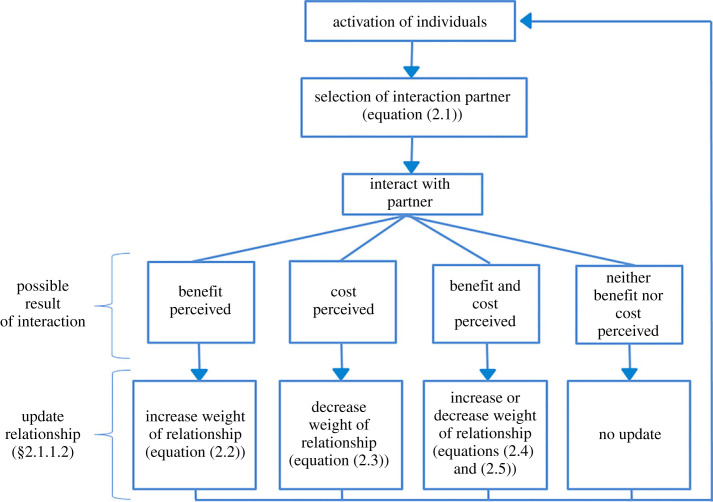
Schematic of the interaction rules occurring at each time step.

Definitions of indices and coefficients used in the model can be found in table 1. A flow diagram illustrating the architecture and processes of the optimal relationship model can be found in figure 2.

**Table 1.** Glossary of parameters.

parameter	definition
*N*	Number of individuals in the group. Group size is set as 10, 30 and 70.
*My-benefits*	The amount of benefits an individual owns. Values are chosen between 0 and 1.
*My-costs*	The amount of costs an individual owns. Values are chosen between 0 and 1.
Social-increase	The percentage increased in the weight between individuals *i* and *j.* The default value is 20%. See §2.1.1.2.
Social-decrease	The percentage decreased in the weight between individuals *i* and *j.* The default value is 20%. See §2.1.1.2.

##### 2.1.1.1. Activation of individuals and interactions

At each time step, all individuals are activated. The probability of individual *i* selecting interaction partner *j* is given by the weight of the relationship (*ϕ_ij_*): the higher the weight of the relationship, the higher the probability that individual *i* selects individual *j* as its interaction partner. The initial probability of interaction $\phi_{01}$ is thus given by the following equation:

2.1 $$\phi_{01}=\frac{1}{N-1},$$

where *N* is the number of individuals in the group and $\phi_{01}$ is the weight of the relationship, which, at initialization, is the same among all group members. Thereafter, the weight of the relationship (and the probability of future interactions with a given partner) is equivalent to the updated weight (see §2.1.1.2).

After an individual selects an interaction partner, there are four possible outcomes of the interaction, the individual perceives: (1) benefits from its partner; (2) costs from its partner; (3) benefits and costs from its partner; or (4) it perceives nothing (figure 2). Each outcome depends on the following probabilities:

1. Probability of perceiving an individual as beneficial but not costly (*PBen*): *PBen* = *My-benefits* of InteractionPartner ∗ (1 – *My-costs* of InteractionPartner)
2. Probability of perceiving an individual as costly but not beneficial (*PCost*): *PCost* = *My-costs* of InteractionPartner ∗ (1 – *My-benefits* of InteractionPartner)
3. Probability of perceiving an individual as beneficial and costly (*PBenCost*): *PBenCost* = *My-benefits* of InteractionPartner ∗ *My-costs* of InteractionPartner
4. Probability of perceiving an individual as neither beneficial nor costly (*Pnone*): *Pnone* = (1 – *My-benefits* of InteractionPartner) ∗ (1 – *My-costs* of InteractionPartner)

To determine the outcome of the interaction, first a random number between 0 and 1 is drawn. This is a process for model stochasticity [40]. The random number is compared with *PBen*, and if the number is less than or equal to that probability, the individual perceives the social interaction as beneficial only. Otherwise, it compares the random number with *PBen* + *PCost*. If the number is less than or equal to the sum of these two probabilities, the individual perceives the interaction as costly only. Otherwise, it compares the random number with *PBen* + *PCost* + *PBenCost*. If the random number is less than or equal to the sum of these three probabilities, the individual perceives the interaction as beneficial and costly—but either more beneficial than costly (when *My-benefits* − *My-costs* > 0) or more costly than beneficial (when *My-benefits* − *My-costs* < 0). Otherwise, it compares the random number with *PBen* + *PCost* + *PBenCost* + *Pnone*. If the random number is less than or equal to the sum of these four probabilities, the individual perceives nothing. Note that *PBen* + *PCost* + *PBenCost* + *Pnone* is equal to 1, and the order of comparison with the random number does not alter the probabilities. At the end of this step, the relationship between *i* and *j* is updated.

It is important to highlight that because the edge weights are uniform in the beginning, individuals interact with a larger number of interaction partners on average than they will later in the simulation. As interactions accumulate, and the weight is continuously updated, individuals learn who holds more benefits and less costs, and thus create their social preferences.

##### 2.1.1.2. Updating relationships

After each interaction, individual *i* will update the weight of its relationship with individual *j* according to whether or not it perceived benefits or costs. If costs were perceived, the weight of interaction between individual *i* and *j* will decrease by 20% of its current value. The 20% subtracted from this relationship is distributed proportionally among the weights of the relationships the agent *i* has with all other group members. Note that this is dampened feedback, meaning that the stronger relationships will be penalized (lose) more than weaker relationships when a cost is perceived. Conversely, if benefits are perceived, the weight of interaction between individual *i* and *j* will increase by 20%. The 20% added to this relationship is obtained from the weights of the relationships the agent *i* has with other group members. This is again dampened feedback, meaning that when benefits are perceived, the reinforcement (gain) during the update is higher for weaker relationships than for stronger relationships. These dampened feedbacks enable a more nuanced assessment of individuals' benefits and costs: by applying stronger penalties to strong relationships and providing stronger reinforcement to weaker ones, we reduce the risk of individuals becoming trapped in local optima. We acknowledge that 20% is an arbitrarily chosen value and that higher or lower values would either increase or decrease the speed at which the network would reach stability. In this way, the weight of relationships is constrained to varying between 0 and 1, and the sum of the weights between individual *i* and all its other group members is kept equal to 1. The updating of relationships is given by the equations in table 2.

**Table 2.** Updating relationship weights. At each time step, after a social interaction, the weight of relationship between individuals *i* and *j* is updated. It depends on the outcome of the interaction whether individual *i* perceives *j* as beneficial, costly, more beneficial than costly, more costly than beneficial or perceives nothing.

outcome of the interaction	weight of relationship (*W*)	
	agent and interaction partner (*W_ij_*)	agent and other group members (*W*_*iz* (*z*≠*j*__)_)
individuals perceive the interaction partner as beneficial	equation (2.2) $W_{ij(t+1)}=W_{ij(t)}+\Delta$	equation (2.2*a*) $W_{iz(t+1)}=W_{iz(t)}\text{–}(W_{iz(t)}\times \mathrm{social}-\mathrm{increase})$
(resulting from *PBen*)	where: $\Delta =\sum_{z\neq j}^{N}Wiz(t)\;\times \mathrm{social}-\mathrm{increase}$	
individuals perceive the interaction partner as costly	equation (2.3) $W_{ij(t+1)}=W_{ij(t)}-\Delta$	equation (2.3*a*) $W_{iz(t+1)}=W_{iz(t)}+\left(\Delta \times \frac{Wiz(t)}{\sum_{z\neq j}^{N}Wiz(t)}\right)$
(resulting from *PCost*)	where: Δ = *W_ij(t)_* × social-decrease	
individuals perceive the interaction partner as more beneficial than costly	equation (2.4) $W_{ij(t+1)}=W_{ij(t)}+\Delta$	equation (2.4*a*) $W_{iz(t+1)}=W_{iz(t)}\text{–}(W_{iz(t)}\times \mathrm{social}-\mathrm{increase}\times \mathrm{dif})$
(resulting from *PBenCost*)	where: $\Delta =\sum_{z\neq j}^{N}Wiz(t)\times (\mathrm{social}-\mathrm{increase}\times \mathrm{dif})$, dif = abs (PBen − PCost)	
individuals perceive the interaction partner as more costly than beneficial	equation (2.5) $W_{ij(t+1)}=W_{ij(t)}-\Delta$	equation (2.5*a*) $W_{iz(t+1)}=W_{iz(t)}+\left(\Delta \times \frac{Wiz(t)}{\sum_{z\neq j}^{N}Wiz(t)}\right)$
(resulting from *PBenCost*)	where: Δ = *W_ij(t)_* × social-decrease × dif, dif = abs (PBen − PCost)	
individuals do not perceive the status of the interaction partner (resulting from *Pnone*)	no update	

#### 2.1.2. Testing conditions

We conducted tests involving 20 different conditions, each representing various combinations of My-benefits and My-costs among group members (figure 1*a*). The values assigned to benefits and costs for each individual were determined based on their unique ID, which ranged from 1 to *N* (with exception for conditions when values are the same for everybody, such as conditions 2 and 6). These assignment functions followed either a linear or power-law pattern (as shown in figure 1*a*), reflecting characteristics of a random network, similar to the linear distribution of components [41]) or analogous to the Pareto distribution, where a small fraction of the population holds a large portion of a given component [42]). Linear distributions were categorized as positive (with values increasing as ID numbers increased) or negative (with values decreasing as ID numbers increased). Power-law distributions were set with a degree exponent of *γ* = 10, resulting in approximately 25% of individuals having values between 1 and 0.1. We tested each of the conditions for theoretical group sizes of 10, 30 and 70 (representative of animal group sizes [33]) to investigate whether the emergent network properties, especially modularity, differed as a function of the number of individuals involved. We ran 20 simulations for each of the 20 conditions tested (400 simulations in total). Each simulation comprised 2000 time steps (= number of interactions per individual). In network depictions of animal societies, it would mean an average of 100 interactions per day, over 20 days.

### 2.2. Data collection and social network analysis

At each time step, we recorded the identities of the interacting individuals as well as the updated values of their relationship weights. These data were divided into 10 time periods, each containing 1000 interactions per individual. From each time period, a matrix of interactions was created including the total number of interactions between each dyad in the group during that time period. These 10 time periods were used for investigating whether social relationships stabilized after a certain point. We observed that the average strength of the relationships did not change after 2000 interactions (electronic supplementary material, figures S1 and S2). Therefore, we used the matrices containing 2000 interactions per individual in our analyses. This dataset gave us the resulting network properties found under each of the conditions. For the social network analysis*,* we chose to estimate network metrics that capture different aspects of network structure at both individual and global levels (table 3).

**Table 3.** Structural properties estimated from the social networks under study. All properties were estimated using the ‘igraph’ package v. 1.0.1 [31] in R v. 3.3.2 [43].

global metrics
Density: the ratio between the number of observed edges and the number of possible edges in the network [36]. Values range from 0 to 1, with 1 reflecting a completely connected network with maximal density. To estimate density, we used the function ‘graph.density’.
Eigenvector centralization: variation in connectedness across nodes in the network [44]. Higher eigenvector centralization values denote a centralized network, where one or a few individuals monopolizes most of the interactions in the network. We estimated eigenvector centralization as $\;\;\;\;\;\;C=\frac{\sum_{i}^{N}\left(C\max-Ci\right)}{\mathrm{Max}\sum_{i}^{N}\left(C\max-Ci\right)},$ where *C* is the centralization index, *Ci* is the centrality for individual *i, C*max is the maximum value of *Ci* across all individuals and $\mathrm{Max}\sum_{i}^{N}\left(C\max-Ci\right)$ refers to what the sum would be under the largest possible centralization of the network. Formula was implemented in the source code.
Newman's modularity: the degree to which a network is divided into differentiable subgroups [45]. We used an eigenvector-based measure that is claimed to be independent of group size. High levels of modularity denote a greater subdivision of the group into subgroups. We estimated Newman's modularity using the function ‘cluster_leading_eigen’.
individual metrics
Betweenness centrality: the number of shortest paths that pass through the considered individual [44]. The more connections that are made through one individual, the greater its value of betweenness becomes. To estimate betweenness centrality, we used the function ‘betweenness'.
Eigenvector centrality: the weighted connectivity of an individual within its network, also considering the weighted connectivity of its neighbours [46]. Individuals tied to central individuals (i.e. those with a high connectivity themselves) should have higher centrality than those connected to less central individuals. To estimate eigenvector centrality, we used the function ‘eigen_centrality’.
Strength centrality: the sum of the link weights connected to each individual [47]. Individuals with the highest strength centralities would be those having strong links, many links or both. We estimated strength centrality using the function ‘strength’.

### 2.3. Global indexes

In order to assess the extent to which the distribution of benefits versus costs affect the development of relationships, we calculated Gini coefficients using values of *My-benefits* (Gini.Ben) and *My-costs* (Gini.Cost) for each condition under study. The Gini coefficient is a statistical measure of distribution, originally used to estimate wealth inequality [48], that has found considerable application in ecology [49–51]. To create a global index that simultaneously considers the variability in distributions of *My-benefits* and *My-costs* across conditions, we simply subtracted Gini.Cost from Gini.Ben,

2.6 Global Index=Gini.Ben −Gini.Cost.

Coefficients ranged from −1 (few individuals monopolizing high values of costs while benefits are more homogeneously distributed among individuals) to 1 (few individuals monopolizing high values of benefits while costs are more homogeneously distributed among individuals), with values equal to 0 indicating perfect equality (Gini.Ben = Gini.Cost).

### 2.4. Statistical analyses

#### 2.4.1. Individual network metrics

We first applied Spearman tests with Bonferroni correction to test whether values of benefits and costs correlated with individual centrality (betweenness, eigenvector and strength; see definitions in table 1). These analyses were important to confirm the predictions of the optimal relationship model, i.e. to show the effects that expressing benefits or costs can have on an individual's position within the social network.

#### 2.4.2. Global network metrics

We applied linear models to test whether the global index and group size correlated with the network properties. We set the retained global indexes as predictor variables in models with the global metrics of network properties (density, modularity and centralization; see definitions in table 3) as response variables. The distribution of all response variables deviated from the Gaussian case, so we applied a square-root transformation (which performed better than log-transformation) to fit the assumptions of the statistical models. For all models, we checked that the assumptions of independent predictors and normally distributed and homogeneous residuals were fulfilled by running a series of diagnostics, including testing for variance inflation and visually inspecting the residuals plotted against fitted values. All analyses were set with the alpha level at 0.05.

Finally, we also looked at the biological importance of model effects through effect sizes. We extracted the standardized coefficients in the regression models and converted it to Cohen's *d* coefficient of effect size [52] using the ‘effectsize’ v. 0.3.0 package in R [53]. For interpreting the extent of an effect, we applied the function ‘interpret_d’.

Alternatively, we also ran principal component analysis (PCA) including the mean values of costs and benefits, in addition to group size, to evaluate their effects on the emergence of network properties. Details on the analysis and results, which are similar to those described in this manuscript, are on the electronic supplementary material.

## 3. Results

Our first set of analyses demonstrates a great diversity of social networks emerging from individual decision rules of social attraction and social avoidance (centralization *x* ± s.d.: 72.11 ± 30.53, modularity: 0.29 ± 0.26, density: 0.33 ± 0.41; figure 1*b*). We first looked at the emergent individual centralities across combined social pressures of benefits and costs in the system. As expected, our correlation analyses demonstrate that individuals with the highest values of *My-benefits* (and thus providing the highest interaction benefits) were those most interconnected in the group (strength: *r* = 0.48, *p* < 0.001; betweenness: *r* = 0.47, *p* < 0.001; eigenvector: *r* = 0.50, *p* < 0.001). On the contrary, and again as predicted, individuals endowed with higher values of *My-costs* exhibited weaker social relationships (strength: *r* = −0.21, *p* = 0.001; betweenness: *r* = −0.43, *p* < 0.001; eigenvector: *r* = −0.26, *p* < 0.001). These results confirm that the agents in our models were behaving as they were designed to behave, being attracted to beneficial partners and avoiding costly partners. We then explored global-level properties of the emergent networks.

### 3.1. Only costly individuals in the system

We found that in conditions 2, 3 and 4, individuals developed social relationships with those endowed with the lowest values of cost. This led to dense (1 ± 0.0008) and non-modular (2.85 × 10^−3^ ± 0.004) networks. Values of eigenvector centralization varied across conditions (33.21 ± 27.44; electronic supplementary material, figure S3), with condition 2 showing a decentralized network (3.98 ± 0.15). When values of costs were distributed differentially across individuals (conditions 3 and 4), we observed the emergence of centralized networks (condition 3: 64.1 ± 6.33; condition 4: 22.6 ± 11.5; electronic supplementary material, figure S3), with condition 3 (i.e. a linear distribution of *My-costs*) presenting the highest values of centralization among the three conditions (*F* = 49.41, *p* < 2 × 10^−16^).

### 3.2. Only beneficial individuals in the system

In conditions 5, 9 and 13, individuals developed social relationships with those endowed with the highest values of benefits. This led to highly centralized (88.38 ± 15.08) and low-density (0.05 ± 0.04) networks (figure 1*b*). The extent of modularity varied across conditions (0.53 ± 0.23; electronic supplementary material, figure S4), with conditions 5 (0.53 ± 0.17) and 9 (0.58 ± 0.20) being more modular than condition 13 (0.41 ± 0.33, *F* = 16.01, *p* < 2 × 10^−16^). The extent of modularity was positively correlated with group size (*r* = 0.81, *p* < 0.05).

### 3.3. Beneficial and costly individuals in the system

**Figure 3.**
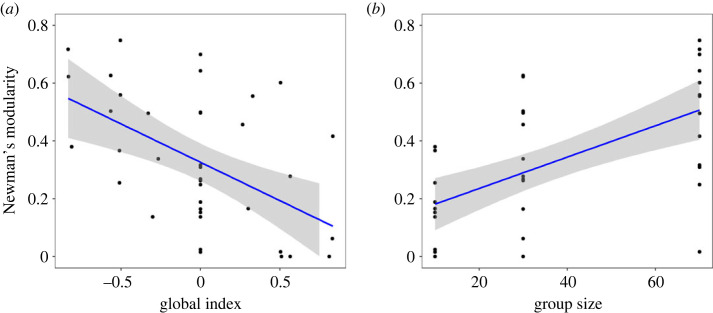
The relationship between modularity, group size and the estimated inequality of costs and benefits in the system (i.e. ‘global index’). (*a*) The negative effect of the global index on the emergence of modular networks. Values in the global index vary from −1 (few individuals monopolizing high values of costs) to 1 (few individuals monopolizing high values of benefits), with values equal to 0 indicating perfect equality (*My-benefits* = *My-costs*). The regression line and its surrounding shaded area show linear model (LM) fit and standard error over replicates. (*b*) The positive relationship between Newman's modularity and group size. Statistical analyses were performed on conditions where individuals hold both benefits and costs in the system (conditions 6–8, 10–12, 14–20).

For conditions in which individuals hold both benefits and costs in the system (conditions 6–8, 10–12, 14–20), we observed centralized (83.28 ± 17.02), modular (0.33 ± 0.23; electronic supplementary material, figure S5) and low-density (0.19 ± 0.29; electronic supplementary material, figure S5) networks. We then looked at whether this variation in structural properties was explained by the group size and the global index, which considers the combined distribution of benefits and costs across individuals. Linear models showed that group size correlated negatively with density but positively with modularity. The global index correlated negatively with modularity (table 4 and figure 3). We found that more modular networks emerged when few individuals had the highest values of costs, and benefits were more homogeneously distributed among several individuals (low global index score). In other words, more modular networks emerged in conditions of low benefit inequality (i.e. a linear distribution; *r_s_* = 0.5, 1 or −1) but high cost inequality (i.e. an exponential distribution), as observed in conditions 8, 12 and 20. We also found that less modular networks emerged when few individuals had the highest values of benefits, but costs were more homogeneously distributed among several individuals (high global index score; figure 3*a*), as observed in conditions 14, 15 and 17. Table 5 provides the effect sizes (Cohen's *d*), which help us to interpret the biological importance of model effects. Finally, we found no effect of group size and global index on network centralization (table 4).

**Table 4.** Relationship between network properties, global index and group size from conditions with both beneficial and costly interactions in the system. Parameter estimates from linear models. Codes are marked as follows: ***p < 0.001.

	estimate	s.e.	*t*-value	Pr(>|*t*|)
*density*				
intercept	0.443	0.027	16.630	<0.001***
global index	0.019	0.033	0.568	0.574
group size	−0.004	0.0006	−6.948	<0.001***
*modularity*				
intercept	0.303	0.048	6.292	<0.001***
global index	−0.319	0.060	−5.320	<0.001***
group size	0.006	0.001	5.173	<0.001***
*centralization*				
intercept	9.191	0.283	32.495	<0.001***
global index	−0.315	0.353	−0.892	0.378
group size	−0.003	0.006	−0.500	0.620

**Table 5.** Comparing effects of global index and group size. Effect size and descriptors for magnitudes follow Cohen's framework [52].

variable	Cohen's *d*		interpretation	
	modularity	density	modularity	density
global index	−1.77	0.20	large	very small
group size	1.72	−2.46	large	large

## 4. Discussion

Our study sheds light on the emergence of divergent network structures in the context of a social trade-off, demonstrating how behavioural variation may result from simple rules of social attraction and social avoidance. Our main findings demonstrate that the accumulation of individual decisions to avoid interactions with highly costly individuals, but that are to some extent homogeneously beneficial, leads to more modular networks. On the contrary, individuals favouring interactions with highly beneficial individuals, but that are to some extent homogeneously costly, leads to less modular networks. Taken together, these results suggest that the underlying distribution of attractive and repulsive forces across individuals can influence network topology. Here, network structure arises from the decisions of individuals aiming to optimize the costs and benefits of social interactions, which is reflective of classical fitness trade-offs.

Immediate behavioural responses of individuals towards costly and beneficial relationships cause detectable changes at the network level [15,21]. A common network property is modularity. There is still not a consensus on the drivers of modularity, but a well-accepted hypothesis is that modular structure emerges to mitigate connection costs [25]. Our study provides theoretical evidence of how modularity initially arises, showing that opposing pressures of social attraction and social avoidance also lead to modular structure. Here, selective pressures that optimize social relationships, avoiding risky interactions while favouring those that might bring the highest benefits, yield modular networks. This has also been observed through evolutionary experiments that aimed to maximize network performance and minimize connection costs [25]. Interestingly, previous studies have suggested that modularity could be paramount in optimizing network efficiency: while low values of modularity favour network efficiency, high values work as a ‘breaker’ [30,54]. The rapid response of individuals that we observed to prevailing conditions leading to varying degrees of modularity suggests that modular networks could allow individuals to navigate costly and beneficial relationships.

From an evolutionary perspective, changes in social structure due to social attraction and avoidance can affect individual fitness in many ways. For example, it has been proposed that successful individuals, considered in terms of those capable of learning about their environment and applying this knowledge in key behaviours such as predator avoidance, are considered reliable interaction partners and receive more affiliative behaviour from others [17]. This places the successful individuals in central positions in the group (e.g. as observed in lemurs, [55,56]), which may confer upon them the role of key information spreaders. Regarding the mechanisms of social avoidance, changes in social rates—either because of lethargy or active avoidance—abound in vertebrate societies [57,58]. A poignant example comes from wild mandrills, in which susceptible individuals avoided grooming conspecifics infected with orofaecally transmitted parasites [19]. According to our model assumptions, individuals generally favoured interactions with individuals providing the highest benefit to cost differential. However, we are unaware of empirical studies that simultaneously consider information and pathogens as opposing pressures driving sociality (but see [6,8] for theoretical studies on beneficial and costly contagion). This has recently been discussed as an important but neglected trade-off in the study of animal societies, including humans [2,59,60].

### 4.1. Comparing baseline conditions

We observed the emergence of modular networks in conditions where benefits and costs existed in the system but also when only benefits existed (conditions 5, 9 and 13). In the latter conditions, modularity is a consequence of individuals being linked to agents with high benefits. In our model, individuals will always prefer to form relationships that bring more benefits than costs. So, individuals will be motivated to seek and interact with agents from which they might get benefits. For example, if two individuals hold benefits in a group of 10, there will be a great probability that the other eight individuals will form social preferences to one of the two individuals holding benefits, which will cause the formation of subgroups. This is more evident in conditions with higher homogeneity in the distribution of benefits in the system (i.e. individuals having a similar value of benefits as in condition 5). When homogeneity is high, individuals perceive benefits from all others, but because of stochasticity, each individual may reinforce their relationship with a different individual over time. So, they end up having preferential interactions with one or two individuals, and every individual may have a different preferred partner. This may lead to the formation of clusters or modular networks.

It is also important to look at conditions with only costs in the system (conditions 2, 3 and 4). In these conditions, individuals had no incentive to form relationships as the main force is that of avoiding costly interactions. This led to the emergence of centralized networks, because individuals preferred interactions with the least risky individuals. In these systems, the model mechanics achieve increases in relationship weight indirectly: by decreasing the weight of relationships with individuals that incur the highest costs of interaction, relationship weights with the least costly individuals increase automatically.

When benefits and costs were included together in the system, these opposing forces played a distinct role in the formation of relationships. Our results indicate that, under this trade-off, the emergence of modular networks was consistent when costs in the system were higher than the benefits observed: the higher the potential for costs and the higher the homogeneity of benefits in the system, the more modular was the network. In conditions where costs and benefits were equivalent (conditions 6, 11 and 16), individuals formed relationships with those providing neither high costs nor high benefits.

### 4.2. Limitations of the study

Our model is simplistic by design. We aimed to build a parsimonious model as a first step towards understanding the emergence of network properties when the system is constrained by opposing pressures of social attraction, driven by potential access to social information, and social avoidance, driven by exposure to costs. Undoubtedly, many other factors affect the development of social relationships in the animal kingdom, including kinship, gender, predation pressure, resource distribution, among others, but building a model including all these factors will probably lead to confounds that would make the results complex and challenging to interpret.

Another limitation of our model is that the mechanics to connect both social attraction and social avoidance is based on the increasing and decreasing of relationship weights with others. This is particularly relevant for conditions where only costs exist in the system: because individuals are forced to interact with others, they will choose those interactions that are less costly, and if all are somewhat costly, they will choose interaction partners at random, which leads to centralized and non-modular networks. If individuals were not forced to interact, it might be that, under the model rules, individuals would avoid interacting at all. Furthermore, our model assumes that some agents hold benefits and/or costs, but it does not account for the dynamics of social transmission (with individuals changing status after an interaction). It is likely that adding such an element of dynamism, with individual status being updated throughout the simulation, would also reinforce certain network structures over others, but it is unclear whether those would be the same as those observed in the current study.

Regardless, such an approach might preclude our ability to test which networks are expected to emerge under a defined set of trade-off conditions (for models on social transmission, see [6,8]). Instead, our model is inspired by studies of behavioural specialization, in which simple rules of interaction based on stable character traits of individuals lead to the formation of complex network topologies [34,61–63]. Our model reinforces the emergence of modular networks even when such simple rules are applied.

## 5. Conclusion

Investigating how individuals respond to specific trade-offs has the potential to deepen our understanding of the mechanisms and selective pressures underlying the development of social relationships. Reflecting the accumulation of individual decisions, modular networks emerged in conditions where interactions are costly with only a few individuals, and beneficial interactions are more homogeneously distributed among all individuals in the group. These results ultimately reveal the importance of looking at the occurrence of opposing pressures with the potential to understand the complexity underlying individual relationships and, consequently, the great diversity in social structure observed across animal societies [64]. We thus hope this study stimulates further investigations into this overlooked trade-off.

## Data Availability

Data are available from the Dryad Digital Repository: http://dx.doi.org/10.5061/dryad.7wm37pw0x [65]. Supplementary material is available online [66].
